# 

**Published:** 1864-09

**Authors:** 


Volume XXI. Number 9.

### THE CHICAGO MEDICAL JOURNAL

De LASKIE MILLER, M. D.,
EPHRAIM INGALS, M. D.,
Editors and Proprietors.

SEPTEMBER, 1864.

CHICAGO:
J. C. W. BAILEY, BOOK AND JOB PRINTER, 130 CLARK STREET.
1864.

